# Atrial Fibrillation Ablation - Are we ready?

**Published:** 2007-08-01

**Authors:** Shantanu Deshpande, Yash Lokhandwala

**Affiliations:** 1 Cardiologist, QECG Services; 2 Arrhythmia Associates

**Keywords:** atrial fibrillation, catheter ablation

Atrial fibrillation (AF) is the most common arrhythmia in day to day practice. It contributes to morbidity in the form of heart failure and stroke and increased overall and cardiovascular mortality [1].   Moreover, no treatment of AF to date has resulted in a lower overall death rate. Anti arrhythmic therapy for AF may also contribute to increased mortality in this subgroup [2]. Even though AFFIRM investigators showed no apparent benefit from rhythm control, these results cannot be generalized to younger patients, more symptomatic patients and those with rheumatic valvular disease. Catheter ablation for the same seems to be an attractive approach.

Catheter ablation for AF is an evolving field. In the late 1990s, a group in Bordeaux demonstrated that the muscle sleeves that surround the pulmonary veins can be very arrhythmogenic and very often supply the triggers that set off atrial fibrillation. This resulted in opening up of new therapeutic avenue that is isolation of the pulmonary veins. Various techniques were described for the same like linear, focal ablation, PV isolation and circumferential antral ablation. Early linear ablation was an empirical version of a "catheter maze," initially limited to the right atrium. With the identification of PV foci, attention was soon directed at these specific targets. Initial ablations have been without 3 D mapping with use of  Lasso or similar catheters. With continuous evolution of  3 D mapping techniques, circumferential antral ablation remains the most common technique used at this time. Variations of it like Wide Area Circumferential Ablation (WACA) or Left Atrial Catheter Ablation (LACA) and Pulmonary Vein Antrum Ablation/Isolation (PVAI) have resulted in better overall outcomes. Till date, however, there has been no standardization of the technique. Even though there is a trend towards better initial success, 6 month cure rates  with new ablative techniques are still inferior to surgical results. Fisher et al [3] in reviewed all publications through 2005 if data included information on technique and 6 month follow up. More than 23000 subjects met the criteria. Cure rate with PVAI was 67 % at 6 months. 25% patients required repeat procedures. They observed trend towards inclusion of patients with permanent and persistent AF with improvement in the various mapping and ablative techniques.

Although there is trend towards reduced fluoroscopy times, still it remains high, i.e. 201 min for PVAI. Complication rates remain high like stroke, repeat procedures, PV stenosis and catastrophic atrio-esophageal fistulae represent real limitation of AF ablative procedures. An atrio-esophageal fistula is a relatively recently recognized complication. Radiofrequency line on the posterior wall of LA can result in thermal injury to the esophagus, as the LA posterior wall is relatively thin measuring about 1.7- 2 mms, whereas radiofrequency ablation can cause 4- 5 mm deep lesions. The esophagus is very sensitive to the thermal injury. This can result in atrio esophageal fistulae which  have very high mortality rates. This complication may be avoided by having simultaneous barium swallow. ***It is a moot question whether it is at all worth undertaking such ablation procedures which have a reported 6% incidence of major complications, including several reported deaths.***

Considering the complexity of the LA anatomy, difficulties in putting confluent anatomical lines in the three dimensional complex LA anatomy, these procedures remain technically challenging. The anatomy can be reconstructed on three-dimensional mapping systems by dragging the catheter around the chamber of interest and building a point-by-point geometry. But acquisition of anatomy  is limited by number of points acquired, also this is a time consuming process needing prolonged fluoroscopy time. Respiratory movement and variations in the PV anatomy also limit the resolution especially of the left veno-atrial junctions.

Radiological investigations like CT scan and MRI can demonstrate LA anatomy very well in all three dimensions. The ability of the CartoMerge Image Integration Module to integrate CT images into the CARTO system can be of advantage by obviating the need of creating an electroanatomic map (EAM). This theoretically should reduce the fluoroscopy times. Merging of images require segmentation of the area of the interest (LA), then alignment with second form of imaging that is EAM. For alignment purposes image has to be oriented in all x y z axes. A minimum of three non-colinear points have to be used for successful registration. Points easily identifiable on fluoroscopy and the CT image at the junction or proximal aspect of at least three different PVs are used.  These points are mapped by EAM and then matched with similar points on the CT images. Various groups have utilized different registration points for the purpose of alignments like PV, aorta [4,5].

Kardos et al describes a new method of  PV isolation using CS ostium as landmark for the registration and merging of the images without any LA mapping in this issue of the journal [6]. They could achieve the isolation of the pulmonary veins in all the patients. Also there was reduction in the fluoroscopy time. However, the study is only of 5 patients. While the method describes the additional lesions in persistent and permanent AF, all their patients had paroxysmal AF. No details of the control group are provided. No clinical follow-up is available to judge the efficacy of the procedure. They have also not specified how the complete isolation of the PVs was validated. With CartoMerge it may be possible to isolate PVs in patients with complex PV anatomy and massively dilated LA. Although there are reports of the successful integration of the images, failures of the integration are mainly due to faulty identification of the registration points.

Even though there is definite progress in catheter ablation of the AF, procedural and periprocedural complications remain high. Moreover immediate success remains 60 -70 % even with best of the techniques, recurrence rate at 6 months remain high (Up to 40 %). Many patients require repeat procedures, and continued anti-arrhythmic therapy. There is no randomized data comparing catheter ablation and medical therapy on hard endpoints. The main obstacles in the therapy are relative inexperience of operators, relative low efficacy of the current techniques and non- availability of randomized data.  It should be used judicially in highly symptomatic patients who have failed drug trial and have significant deterioration of the quality of life.

## References

[R1] Wolf PAS, Mitchell JB, Baker CS (1998). Impact of atrial fibrillation on mortality, stroke, and medical costs. Arch Intern Med.

[R2] The Atrial Fibrillation Follow-up Investigation of Rhythm Management(AFFIRM) Investigators (2002). A comparison of rate control and rhythm control in patients with atrial fibrillation. N Engl J Med.

[R3] Fisher JD, Spinelli MA, Mookherjee D (2006). Atrial fibrillation: reaching the mainstream. Pacing Clin Electrophysiol.

[R4] Reddy VY, Malchano ZJ, Neuzil P (2005). Early clinical experience with Carto-merge for integration of 3D-CT imaging with real-time mapping to guide catheter ablation of atrial fibrillation. Heart Rhythm.

[R5] Kistler PM, Earley MJ, Harris S (2006). Validation of three-dimensional cardiac image integration: Use of integrated CT image into electroanatomic mapping system to perform catheter ablation of atrial fibrillation. J Cardiovasc Electrophysiol.

[R6] Kardos Attila, Foldesi Csaba, Ladunga Karoly (2007). Pulmonary Vein Isolation Without Left Atrial Mapping. Indian Pacing and Electrophysiology Journal.

